# Whole-Genome Sequencing of RSV and Phylogeographic Assessment of Viral Importations into Russia

**DOI:** 10.3390/v18080901

**Published:** 2026-08-15

**Authors:** German V. Roev, Ekaterina V. Pimkina, Dmitry V. Svetlichnyy, Arina V. Peresadina, Maksim I. Nadtoka, Kamil F. Khafizov, Vasiliy G. Akimkin

**Affiliations:** Central Research Institute of Epidemiology, 111123 Moscow, Russia; roevherman@gmail.com (G.V.R.); tsibina@cmd.su (E.V.P.); dim_stalker@mail.ru (D.V.S.); peresadina@cmd.su (A.V.P.); nadtoka@cmd.su (M.I.N.); vgakimkin@yandex.ru (V.G.A.)

**Keywords:** respiratory syncytial virus, RSV, NGS, next-generation sequencing, genomic surveillance, phylogeography, molecular epidemiology, viral introductions, Russia

## Abstract

Lower respiratory tract infections caused by the respiratory syncytial virus (RSV) pose a major global public health challenge. The use of next-generation sequencing technologies enables detailed monitoring of viral genetic variability, which is crucial for evaluating the efficacy of immunoprophylactic measures. In this study, whole-genome sequencing of RSV was performed on 106 samples collected in the Russian Federation between September 2021 and April 2025. Three NGS platforms were employed: Illumina MiSeq, Oxford Nanopore Technologies MinION, and Qitan Tech QNome-3841. Using discrete phylogeographic methods, we estimated a minimum of 45 introduction events into Russia for RSV-A and 39 for RSV-B among the genomes included in the analysis. Most events were represented by a single Russian genome. These results indicate recurrent introductions of RSV into Russia from abroad. Given the limited genomic sampling available, most of these introductions were not associated with detectable transmission within the country.

## 1. Introduction

Respiratory syncytial virus (RSV) is one of the leading causes of lower respiratory tract infections worldwide [1]. According to a global meta-analysis, children under 5 years are the age group most vulnerable to RSV [2]. Specifically, due to acute respiratory viral infections caused by RSV, 3.6 million hospitalizations of children aged 0 to 5 years were reported worldwide in 2019, of which 1.4 million cases were reported among infants aged 0 to 6 months [2]. In addition to Palivizumab, which was approved for use in 1998, other prophylactic and/or therapeutic agents of RSV-associated infections have emerged in recent years: the monoclonal antibodies Nirsevimab and Clesrovimab, as well as three vaccines (Abrysvo, Arexvy, mRESVIA) [3,4,5]. Given that the pathogen’s ongoing evolution may reduce the efficacy of these agents, continuous monitoring of the virus’s genetic variability is essential [6].

RSV belongs to the species Orthopneumovirus hominis of the family Pneumoviridae. Two antigenic subgroups of a virus are distinguished: RSV-A and RSV-B [7]. The viral genome consists of a negative-sense single-stranded RNA molecule approximately 15,200 nucleotides in length [8]. The genome contains 10 genes that encode 11 proteins [9]. Among these, glycoproteins G and F are of greatest interest to researchers, as they facilitate the attachment of the viral particle to the cell and its subsequent entry, respectively [9]. These glycoproteins, which are present in large quantities on the surface of the viral envelope, exhibit varying degrees of variability. Thus, the amino acid sequences of glycoprotein G differ significantly between the two subgroups, RSV-A and RSV-B (53% identity), whereas for glycoprotein F, this figure is higher, at approximately 89% [10].

The emergence of new experimental protocols and the development of next-generation sequencing (NGS) technologies make it possible to obtain whole-genome data for a large number of samples. Monitoring is important for tracking mutations in key viral proteins, as their accumulation can reduce the effectiveness of immunoprophylactic measures. In recent years, studies have emerged demonstrating the applicability of various sequencing platforms—using both short and long reads—for this purpose [4,11]. Obtaining sequences of complete viral genomes also facilitates more reliable phylogenetic analysis, which, from an epidemiological standpoint, allows for more accurate identification of viral introduction events in a given region. Reconstruction of RSV transmission routes using phylogenetic methods has indicated that the main hubs in the global virus distribution network are the United Kingdom, the United States, and Australia [12]. In particular, it has been shown that the United Kingdom and Australia were the primary sources of imported RSV-A cases in Russia [12].

Similar analyses of the spread of respiratory syncytial virus in the Russian Federation have primarily been conducted using the G-protein gene sequence, rather than the full genome [13]. One of the main reasons for this is the limited number of whole-genome sequences of Russian virus isolates in international databases (such as GISAID) [14].

The aim of this study is to sequence the complete genomes of RSV-A and RSV-B from samples collected in the Russian Federation using various NGS platforms (Illumina MiSeq, ONT MinION, and Qitan Tech QNome-3841) and, using discrete phylogeographic methods, to estimate the number of viral introduction events represented among the available Russian RSV genomes.

## 2. Materials and Methods

### 2.1. Clinical Material

The study included 106 nasopharyngeal and oropharyngeal swabs from patients with laboratory-confirmed respiratory syncytial virus infection, referred from healthcare facilities in Moscow, the Moscow region and the Kabardino-Balkarian Republic, collected between September 2021 and April 2025. Patient age ranged from 0 to 73 years, with a predominance of individuals under 18 years of age. Nasopharyngeal and/or oropharyngeal swabs were collected from each patient. When both swabs were obtained, they were combined in a single transport medium and processed as one composite sample. In some cases, only a nasopharyngeal or an oropharyngeal swab was available. The available records did not permit retrospective differentiation between combined and single-site specimens. Of the 106 clinical samples, 35 were sequenced on the Oxford Nanopore Technologies (ONT) MinION platform, 25 on the Qitan Tech QNome-3841 platform, and 46 on the Illumina MiSeq platform. The samples were sequenced in several batches over the study period, and the choice of platform depended on the availability of instruments and reagents at the time of processing. Platform assignment was not based on sample characteristics and was not intended for a direct cross-platform comparison.

### 2.2. Nucleic Acid Extraction and Reverse Transcription

Nucleic acid extraction was performed using the RIBO-prep RNA/DNA extraction kit (AmpliSens, Moscow, Russia) in accordance with the manufacturer’s instructions. Reverse transcription was carried out using the Reverta-L reagent kit (AmpliSens, Russia) according to the manufacturer’s protocol.

### 2.3. Amplification of RSV Whole Genomes

A modified primer panel was employed, developed on the basis of the ARTIC protocol (https://github.com/artic-network/artic-rsv/, accessed on 5 April 2024). Primers were selected from the original set such that the target amplicon length fell within the range of 1000–1200 bp. Primer sequences and their concentrations are provided in Table S1 (Supplementary Materials).

PCR was performed on cDNA derived from each sample in two separate reactions. The reaction mixture contained: 5 µL of cDNA, 12.5 µL of Q5 High-Fidelity 2× Master Mix (New England Biolabs, USA), 2 µL of the corresponding primer pool (2.5 µM), and 5.5 µL of deionized water. Amplification was carried out under the following cycling conditions: 98 °C for 30 s (1 cycle); 98 °C for 10 s, 63 °C for 30 s, 72 °C for 2 min (35 cycles); and 72 °C for 2 min (1 cycle).

### 2.4. Library Preparation for Paired-End Sequencing on the Illumina MiSeq Platform

Amplicons generated from the two primer pools were combined in equal proportions and purified using AMPure XP magnetic beads (Beckman Coulter, Brea, CA, USA) at a ratio of 0.7× (beads to sample). Following purification, the amplicons were fragmented using a Covaris ultrasonicator (Covaris, Woburn, MA, USA). End repair and dA-tailing were performed using the NEBNext Ultra II End Repair/dA-Tailing Module (New England Biolabs, Ipswich, MA, USA), after which a Y-adapter compatible with the IDT for Illumina Nextera XT Index Kit v2 (Sets A–D) was ligated using the NEBNext Ultra II Ligation Module (New England Biolabs, USA). The fragments were then re-purified using magnetic beads at a ratio of 1.2× (beads to sample). Sequencing libraries were constructed by indexing PCR using NEBNext Ultra II Q5 Master Mix (New England Biolabs, USA), and the resulting libraries were purified using magnetic beads at a ratio of 1.1× (beads to sample). The libraries were subsequently pooled in equimolar proportions and subjected to double size selection. Library quality was assessed using an Agilent 2100 Bioanalyzer (Agilent Technologies, Santa Clara, CA, USA). Sequencing was performed on a MiSeq instrument (Illumina, San Diego, CA, USA) using the MiSeq Reagent Kit v3 (600 cycles, paired-end) (Illumina, USA).

### 2.5. Library Preparation for Sequencing on the Oxford Nanopore Technologies Platform

Amplicon pooling and purification were carried out as described above. Library preparation was performed according to the manufacturer’s instructions for the Native Barcoding Kit 96 V14 (SQK-NBD114.96) (Oxford Nanopore Technologies, Oxford, UK). The prepared libraries were pooled, purified using magnetic beads at a ratio of 0.5× (beads to sample), and ligated with the Native Adapter included in the Native Barcoding Kit 96 V14 using the NEBNext Quick Ligation Module (New England Biolabs, USA). A final bead-based purification was performed at a ratio of 0.4× (beads to sample). Sequencing was conducted on a MinION device (Oxford Nanopore Technologies, UK) using an FLO-MIN114 flow cell (Oxford Nanopore Technologies, UK).

### 2.6. Library Preparation for Sequencing on the Qitan Tech Platform

Amplification products were combined in equal proportions and purified using AMPure XP magnetic beads (Beckman Coulter, USA) at a ratio of 0.7× (beads to sample). Library preparation was performed according to the manufacturer’s instructions for the Ligation Native Barcoding Kit (QNLK-48 V1.4) (Qitan Tech, Chengdu, China). Following purification, amplicon end repair and barcode ligation were carried out using the Ligation Native Barcoding Kit. The prepared libraries were pooled, purified using magnetic beads, and ligated with the Sequencing Adaptor included in the Ligation Native Barcoding Kit. A final bead-based purification was performed at a ratio of 0.4× (beads to sample). Sequencing was conducted on a QNome-3841 instrument (Qitan Tech, China) using the DNA Sequencing Kit (QDS v1.1) (Qitan Tech, China) and a QCell-384 flow cell (Qitan Tech, China).

### 2.7. Bioinformatic Analysis of Sequencing Data

For long-read datasets (ONT and Qitan), read trimming was performed using Chopper v0.11.0 with the parameters --trim-approach trim-by-quality --cutoff 5 -l 40 -q 10 [15]. For Illumina data, read trimming was carried out using Trimmomatic v0.39 with the parameters ILLUMINACLIP:NexteraPE-PE.fa:2:30:10 SLIDINGWINDOW:4:15 MINLEN:40 [16]. Primer sequences were subsequently trimmed from all three datasets using cutadapt v4.9, with primer sequences specified as non-internal adapters [17]. For ONT data, draft consensus genomes were generated using a variant-based Medaka workflow. First, inference was performed from the sorted BAM files using Medaka v2.1.1 (medaka inference), followed by variant calling against reference genome with medaka vcf. The resulting VCF files were further annotated using medaka tools annotate. Only variants marked as PASS were retained, normalized with bcftools norm, and filtered to remove low-depth calls with DP < 30. Additional quality filtering was applied to exclude SNPs and MNPs with QUAL < 5, and indels or other variant types with QUAL < 10. The filtered VCF files were used to generate draft consensus sequences with bcftools consensus. The resulting draft consensus sequences were then used as references for a second round of read mapping with minimap2 v2.30-r1287 (map-ont preset, secondary = no options) to determine sequencing depth and identify positions with insufficient coverage [18]. Positions with coverage below 30× were masked with Ns using bedtools maskfasta v2.25.0 [19]. For Qitan data, reads were mapped to the reference genome using minimap2 (map-ont preset, secondary=no option). Variant calling was then performed using BCFtools v1.22 (bcftools mpileup --fasta-ref|bcftools call --ploidy 1 -Ou -mv|bcftools norm -m-any), followed by variant filtering (bcftools view -i “INFO/DP > 29”|bcftools filter -e ‘(TYPE = “snp” && QUAL < 20) || (TYPE = “indel” && QUAL < 50)’’) [20]. Draft consensus sequences were generated using bcftools consensus. Reads were subsequently remapped to the draft consensus sequences with minimap2, and positions with coverage below 30× were masked with Ns. For Illumina data, the assembly workflow was analogous to that used for Qitan data, with the exception that read mapping was performed using bowtie2 v2.5.5 (--local option), variants were filtered (bcftools view -i “INFO/DP > 9” | bcftools filter -e ‘(TYPE = “snp” && QUAL < 20) || (TYPE = “indel” && QUAL < 50)’), and the minimum coverage threshold for masking was set to 10× [21]. BAM files sorting was performed using samtools v1.22.1 [20]. Final consensus sequences from all sequencing platforms were manually curated by inspecting read alignments in IGV [22]. Low-confidence sites and regions, including regions with inconsistent read support or local coverage anomalies, were masked with Ns before downstream analyses.

For sample genotyping, BLASTn and Nextclade were integrated into a unified pipeline implemented in Python [23,24]. The resulting code was incorporated into the VGARus, a genomic surveillance platform previously described for SARS-CoV-2, which has since been extended to include, among other updates, RSV subtype and clade classification [25]. The subtype of each sequence was determined using BLASTn, and clade assignment was performed using Nextclade. Subtype and clade assignment were used only for preliminary classification of sequences into RSV-A and RSV-B datasets. Subsequent phylogenetic and phylogeographic analyses, including the inference of introduction events, were performed using whole-genome alignments.

### 2.8. Analysis of Viral Introduction Events

RSV-A and RSV-B genomic sequences obtained through the present study were selected for analysis. All analyses were performed separately for each of the two viral subtypes. Additionally, whole-genome sequences from the international databases GISAID and NCBI GenBank were incorporated, provided they included information on the date and country of sample collection [26,27]. Among the sequences generated in this study, only genomes with more than 80% genome-length coverage were retained for analysis. Among publicly available sequences from GenBank and GISAID, only genomes with at least 90% genome-length coverage were included. For samples with only the year and month of collection specified, the first day of the respective month was assigned as the collection date. If only the year was available, July 1 of the corresponding year was used as the collection date.

To minimize redundancy in the dataset, sequences from GISAID and GenBank were deduplicated. Genomes with 100% pairwise identity across their full length were considered potential duplicates. If such sequences originated from the same country and had collection dates differing by no more than two weeks, they were merged into a single cluster. One representative sequence was retained per cluster, corresponding to the earliest collection date. If a given genomic sequence was assigned to multiple clusters, only the representative with the earliest collection date was carried forward for analysis.

Separate sequence datasets for RSV-A and RSV-B were assembled for Russia, comprising sequences generated in the present study alongside sequences retrieved from GISAID and GenBank. To further reduce the number of international sequences, an additional filtering step was applied. For further sequence filtering, all genomes were aligned using Nextclade, and pairwise nucleotide identity was calculated. For each Russian sample, up to 50 closest foreign sequences were selected, requiring >97% nucleotide identity and >92% coverage of the Russian query sequence [24]. An additional subsampling step was applied to ensure balanced temporal and geographic representation, with a maximum of 50 sequences per country per year retained.

Multiple sequence alignments were constructed using NextClade [24]. Sequence EPI_ISL_19871847 was excluded from the analysis because of anomalous alignment patterns with unusually long insertions. To improve the reliability of phylogenetic reconstruction, poorly covered terminal regions were trimmed from the alignments (trimmed lengths: 85 nt at the 5′ end and 257 nt at the 3′ end for RSV-A; 55 nt at the 5′ end and 53 nt at the 3′ end for RSV-B). Phylogenetic trees were inferred using FastTree v2.1.11 (-nt -gtr -gamma) [28]. Temporal signal assessment and tree rooting were performed in TempEst [29]. Sequences with a residual value exceeding 0.05 in TempEst were excluded, after which alignments and trees were reconstructed.

Recombinant sequences were identified using RDP5 (highest acceptable *p*-value: 0.01; sequence type: linear), with recombination events required to be supported by three or more methods [30]. Following exclusion of recombinants, alignments and trees were rebuilt as described above. For root-to-tip regression analysis, the optimal root was selected in TempEst using the heuristic residual mean squared option, and the data were exported as a table [29]. Regression plots were generated using the linregress function from the scipy.stats package (v1.11.2) in Python 3.10. Identifiers of the final sets of foreign sequences are listed in Supplementary Table S3 and File S1. Identifiers of the sequences obtained in this study are listed in Supplementary Table S2.

Temporal calibration and rooting of the inferred trees were performed using the Augur pipeline from the Nextstrain project (augur refine --tree tree.newick --alignment alignment.fasta --metadata metadata.csv --timetree --output-tree augur_tree.newick --output-node-data nodes.json --seed 8) [31]. Ancestral state reconstruction at internal nodes was carried out using TreeTime (treetime mugration --tree augur_tree.newick --states metadata.csv --attribute location --outdir mugration --confidence --rng-seed 8) [32]. The metadata.csv file contained binary location attributes for each sample, coded as “Russia” or “Non-Russia”. TreeTime assigned to each internal node a probability of belonging to either the “Russia” or “Non-Russia” class. A viral introduction event was defined as a transition from a parental node assigned to “Non-Russia” (with probability >70%) to a descendant node assigned to “Russia” (with probability >70%). Along each root-to-tip path, only the earliest such introduction event was counted.

The robustness of viral introduction count estimates was evaluated using three approaches. First, the number of introductions was assessed on the original trees using alternative probability thresholds for node classification into the “Russia” and “Non-Russia” categories: in addition to the primary threshold of 70%, values of 60% and 80% were also applied. Second, phylogenetic trees were additionally inferred using IQ-TREE v3.0.1 (with the -m GTR+G option), and the introduction analysis was repeated on these trees [33]. Third, subsampled datasets of reduced size were employed. Random subsamples were drawn from the original international sequence dataset at 50%, 60%, 70%, 80%, and 90% of the original size, with 10 replicates generated for each subsampling level. All available Russian sequences were retained in full for each replicate. The number of viral introduction events was then quantified across all subsampled datasets.

For RSV-A and RSV-B separately, ancestral nucleotide sequences were reconstructed at internal nodes of the phylogenetic trees using augur ancestral. Amino acid substitutions in the F and G proteins were subsequently inferred using augur translate [31]. Two-sided Fisher’s exact tests and Holm correction for multiple comparisons were performed in Python 3.10 using the scipy.stats and statsmodels.stats.multitest modules, respectively. Adjusted *p*-values below 0.05 were considered statistically significant.

In addition, the F protein sequences for all Russian RSV samples were screened for amino acid substitutions previously reported to reduce susceptibility to nirsevimab and palivizumab. The list of resistance-associated substitutions, compiled with reference to the ViralZone RSV resource (https://viralzone.expasy.org/11605, accessed on 4 August 2026), is provided in Supplementary Table S5 [34,35,36,37,38,39,40,41].

## 3. Results

A phylodynamic approach was applied to analyze viral introduction events of RSV into Russia from abroad. The rationale of this method involves constructing a phylogenetic tree from a combined dataset of sequences originating from Russia and from other countries, followed by ancestral state reconstruction at internal nodes and enumeration of introduction events. The state variable in this context is a binary attribute indicating whether a given sequence originates from Russia or not.

**Figure 1:**
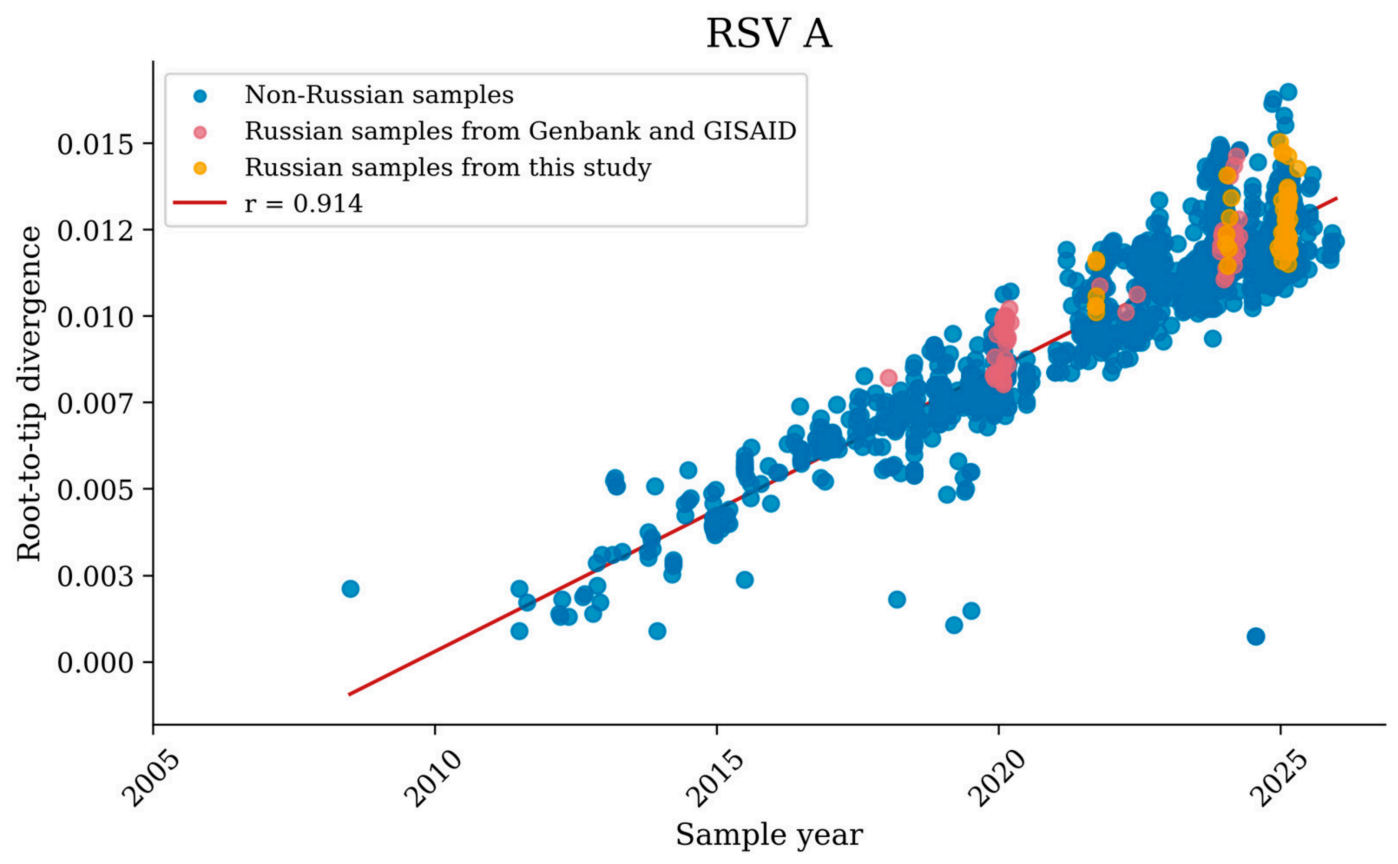
Temporal signal analysis for RSV-A whole-genome sequences. Each point represents one RSV-A genome included in the dataset. The x-axis shows the sampling year, and the y-axis shows root-to-tip divergence, defined as the distance along the maximum likelihood tree from the inferred root to each terminal sequence. The red line indicates the linear regression between sampling date and root-to-tip divergence. The correlation coefficient is shown in the legend.

**Figure 2:**
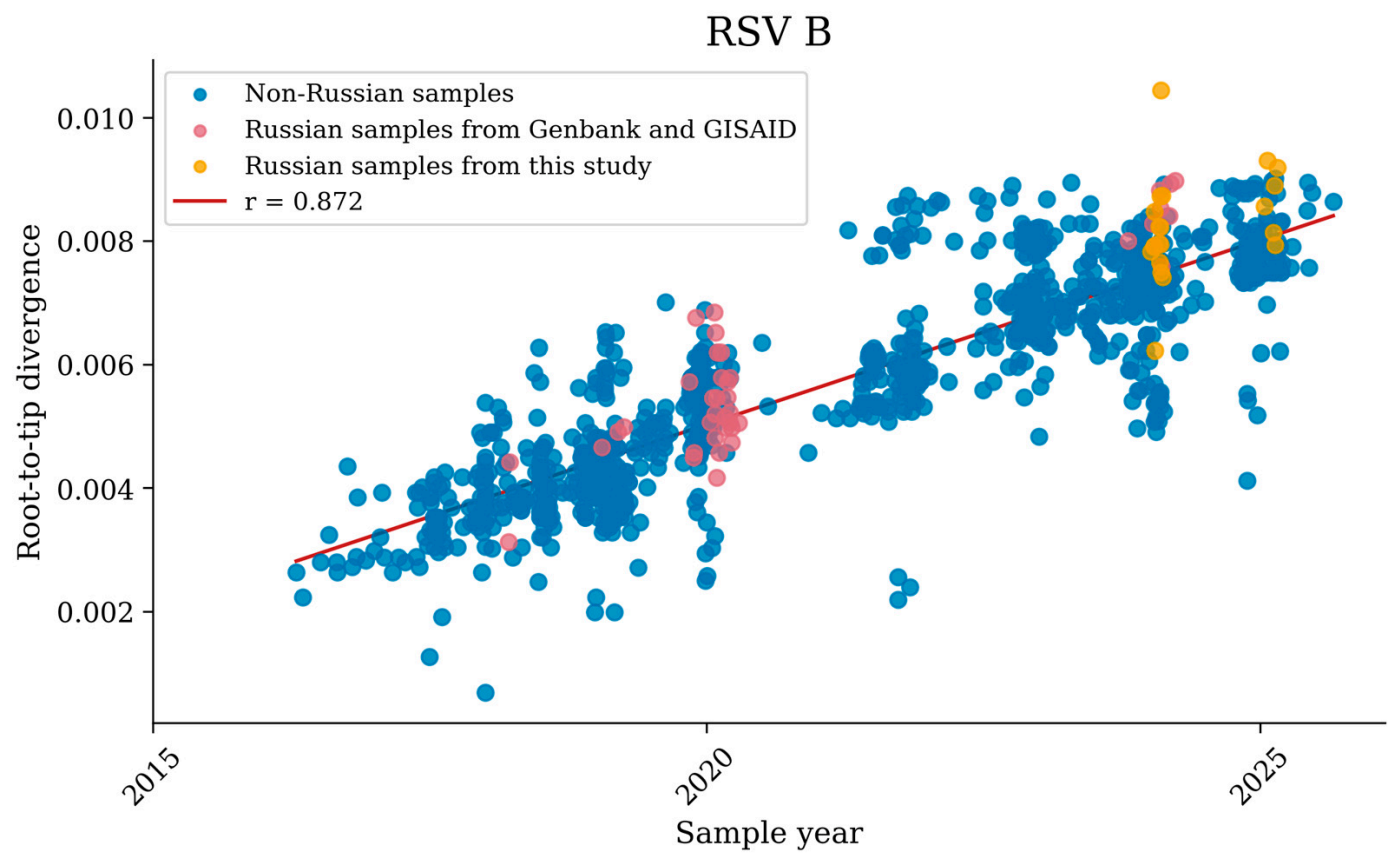
Temporal signal analysis for RSV-B whole-genome sequences. Each point represents one RSV-B genome included in the dataset. The x-axis shows the sampling year, and the y-axis shows root-to-tip divergence, defined as the distance along the maximum likelihood tree from the inferred root to each terminal sequence. The red line indicates the linear regression between sampling date and root-to-tip divergence. The correlation coefficient is shown in the legend.

Following quality filtering of the genomic sequences generated in the present study, 53 sequences were retained for RSV-A and 21 for RSV-B. After incorporating sequences from GISAID and GenBank, temporal signal analysis was performed, revealing a strong correlation (correlation coefficient more than 0.87 for both viral subtypes) between sampling date and root-to-tip distance (Figure 1 and Figure 2). No recombination was detected among analyzed sequences, which is consistent with the generally low frequency of recombination reported for RSV [42].

The final dataset used for maximum likelihood phylogenetic reconstruction comprised 1655 RSV-A genomic sequences, of which 108 were of Russian origin. Similarly, 1166 sequences were used for RSV-B, of which 60 were from Russia.

Phylogeographic analysis revealed multiple viral introduction events into Russia from abroad. These could be classified into two groups: singleton introductions, represented by a single Russian sequence, and cluster introductions, represented by two or more Russian sequences. For RSV-A, a total of 45 introduction events were identified, of which 28 were singletons; for RSV-B, 39 introduction events were identified, of which 28 were singletons. Although singleton introductions constituted the majority of inferred introduction events, they accounted for a minority of the Russian sequences analysed: 28 of 108 sequences (26%) for RSV-A and 28 of 60 sequences (47%) for RSV-B. The remaining sequences were either assigned to cluster introductions or were not assigned to any introduction event, as no parent–descendant transition along the corresponding root-to-tip path satisfied the probability thresholds used to define an introduction.

**Figure 3:**
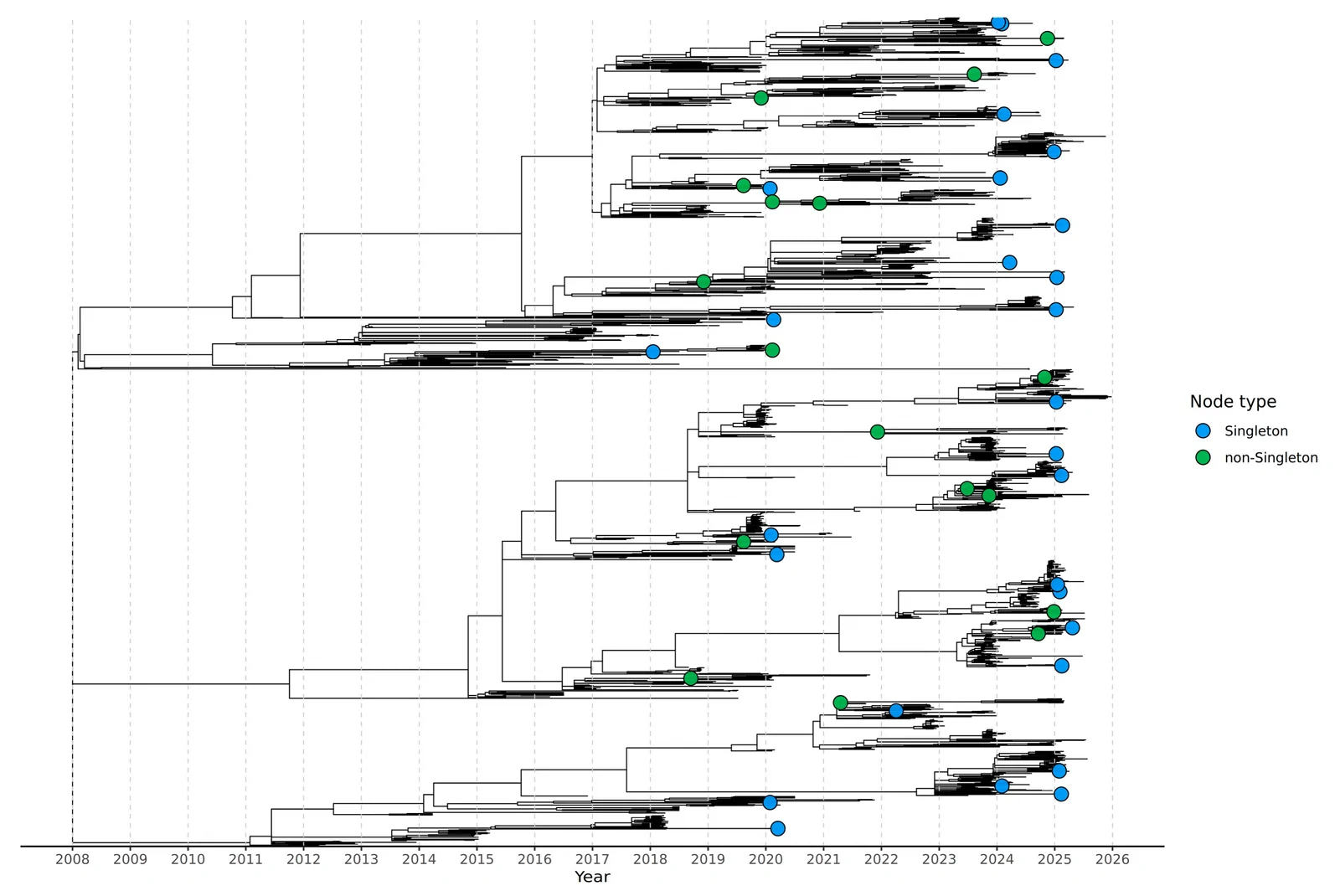
RSV-A phylogenetic tree. Nodes corresponding to singleton introduction events (represented by a single Russian sequence) are highlighted in blue. Cluster introduction events are indicated in green.

**Figure 4:**
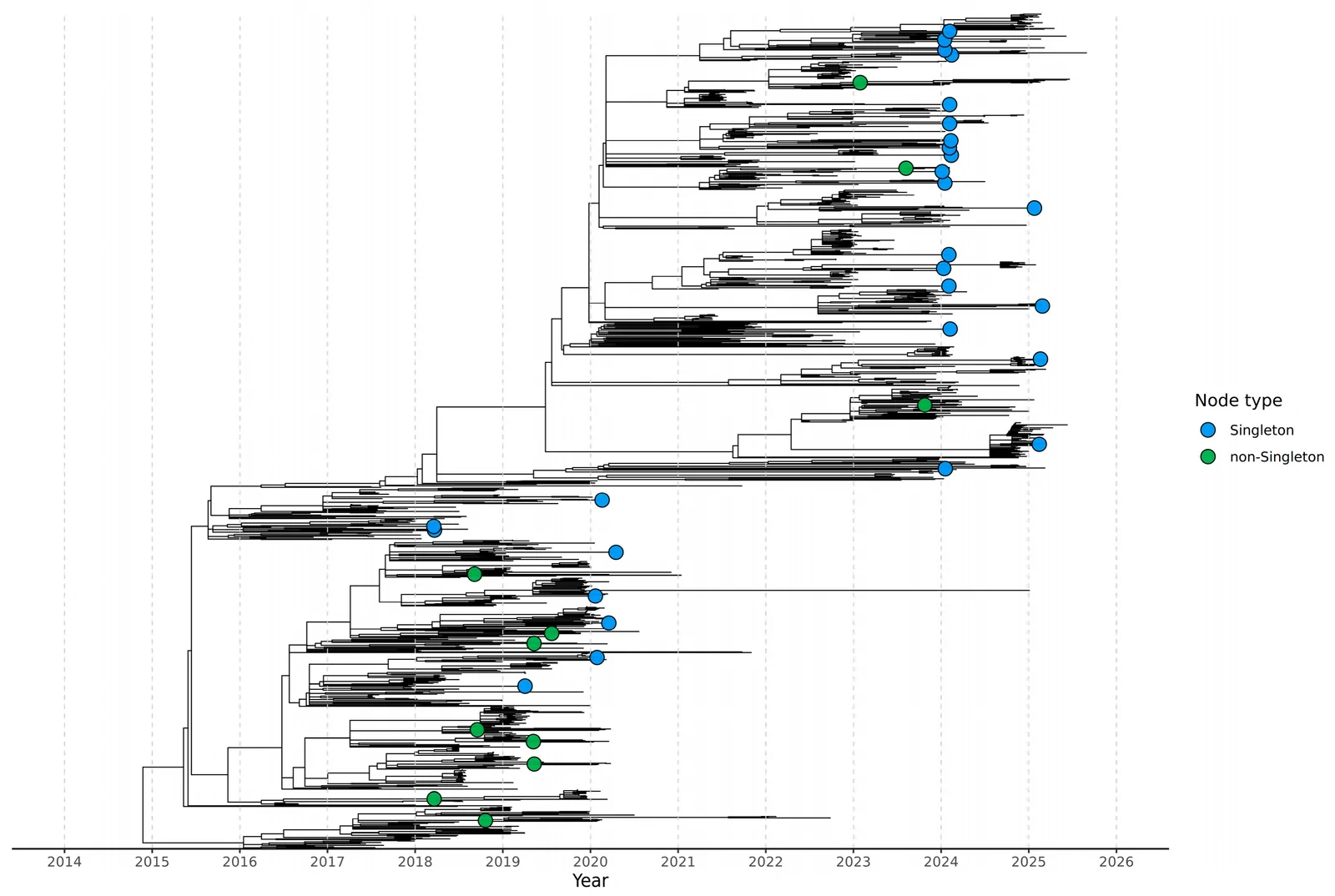
RSV-B phylogenetic tree. Nodes corresponding to singleton introduction events (represented by a single Russian sequence) are highlighted in blue. Cluster introduction events are indicated in green.

The same curated RSV-A and RSV-B datasets that passed temporal signal assessment were then used for time-scaled phylogenetic reconstruction. Thus, Figure 1 and Figure 2 provide the temporal signal assessment supporting molecular-clock-based phylogenetic analysis, whereas Figure 3 and Figure 4 show the corresponding time-scaled phylogenies used to identify singleton and cluster-associated introduction events.

**Figure 5:**
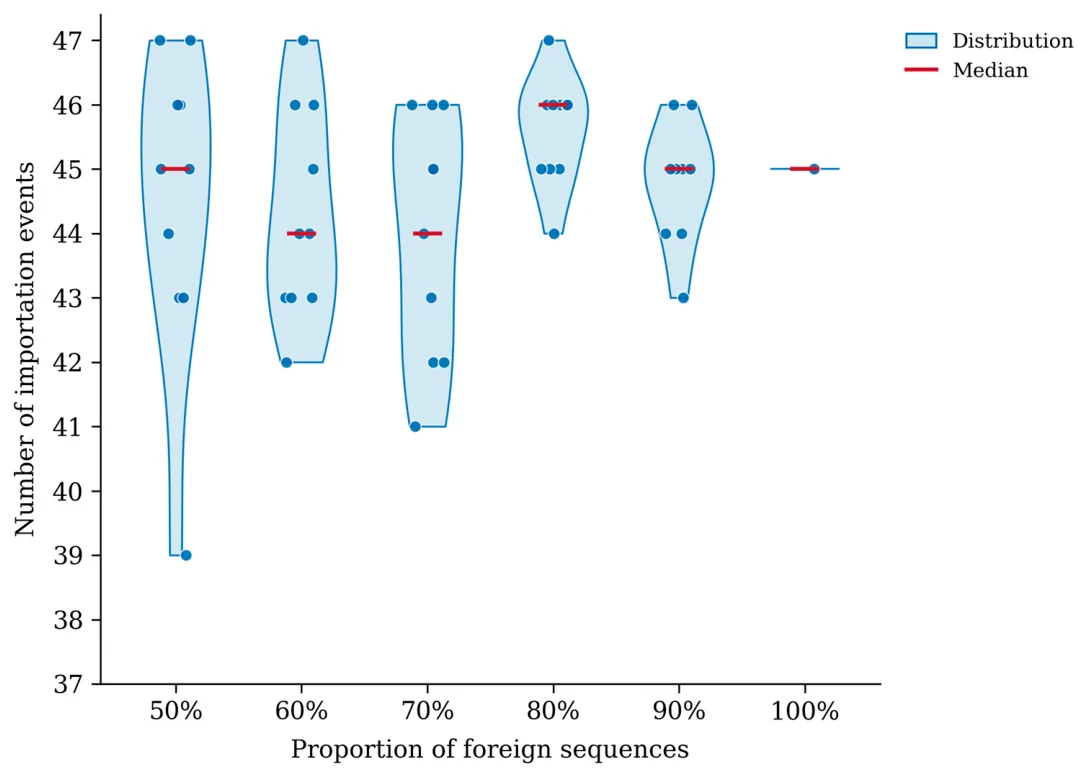
Effect of the number of international sequences on the estimated number of viral introduction events for RSV-A.

**Figure 6:**
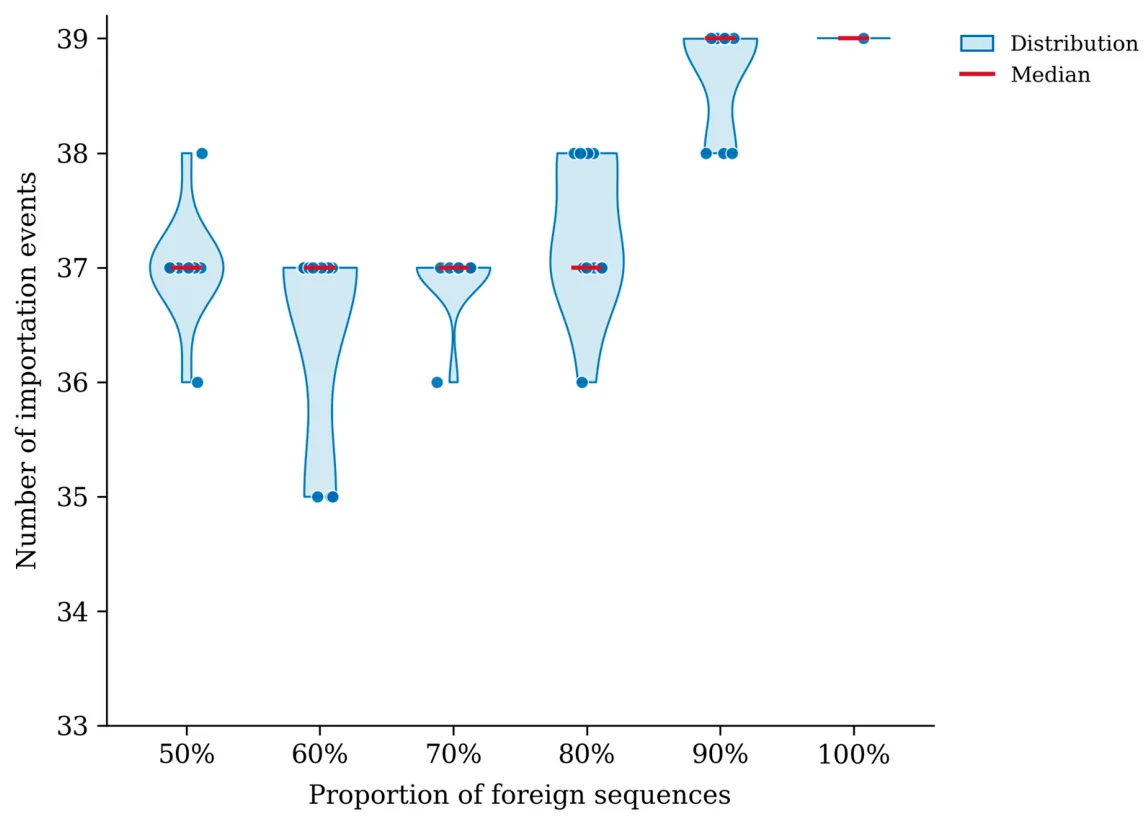
Effect of the number of international sequences on the estimated number of viral introduction events for RSV-B.

The number of international genomic sequences included in the analysis had little effect on the estimated number of viral introduction events. For RSV-A, the median number of introductions ranged from 44 to 46, while for RSV-B it ranged from 37 to 39, across subsampling levels ranging from 50% to 100% of the international sequence dataset (Figure 5 and Figure 6). This indicates that the results are robust to variation in the number of non-Russian sequences included. Varying the probability threshold for assigning internal nodes to the “Russia” state, as well as the use of an alternative maximum likelihood tree inference method IQ-TREE 3 (Table 1 and Table 2), likewise had no substantial effect on the results.

The results obtained indicate multiple introduction events of RSV into Russia, most of which were not followed by detectable onward transmission. The robustness of these findings to variation in the proportion of international sequences included, the probability thresholds applied for classifying internal nodes as Russian, and the phylogenetic reconstruction method employed supports the reliability of the reported estimates.

To assess whether the presence of amino acid substitutions in the F and G proteins was associated with the formation of detectable transmission clusters, we compared cluster introductions, comprising multiple Russian sequences, with singleton introductions. For RSV-A and RSV-B separately, two-sided Fisher’s exact tests were used to compare the proportions of introduction-associated branches carrying at least one reconstructed amino acid substitution in the F or G protein. None of the four comparisons remained statistically significant after Holm correction for multiple testing (Supplementary Table S4a). The only substitutions observed in more than one cluster introduction were G:T138I in RSV-A, which occurred in two cluster introductions, and G:T279A in RSV-B, which occurred in two cluster introductions and two singleton introductions. Thus, no statistically significant association was identified between the presence of F- or G-protein amino acid substitutions and introduction type (adjusted *p* > 0.05 for all comparisons). Reconstructed amino acid substitutions in the F and G proteins on introduction-associated branches are presented in Supplementary Table S4b,c for RSV-A and RSV-B, respectively.

Among the Russian sequences analysed, a single RSV-B genome retrieved from GISAID (EPI_ISL_19437722) carried the F:N201S substitution, previously associated with reduced susceptibility to nirsevimab; no other resistance-associated substitutions in the nirsevimab binding site were detected, and none of the sequences carried substitutions associated with resistance to palivizumab [37].

## 4. Discussion

In Russia, RSV infection is one of the major causes of hospitalization due to lower respiratory tract infections among young children, which is consistent with global epidemiological data [14,43]. The COVID-19 pandemic was associated with a marked disruption of RSV seasonality and a sharp decrease in detected RSV cases in Russia from April 2020 to March 2021 [44]. In the following post-pandemic seasons, changes in the circulating RSV subtype structure were observed. In 2022–2023, RSV-B replaced RSV-A as the dominant subtype, whereas in the 2023–2024 season the emergence of the RSV-A clade A.D.5.2, apparently new to Russia, was likely associated with a sharp increase in RSV circulation [13]. These observations highlight the importance of genomic surveillance and monitoring of international introductions of genetically distinct RSV variants, as limited pre-existing population immunity to newly introduced lineages may facilitate their rapid spread.

Phylogenetic approaches have been applied to investigate viral introduction events into specific regions, and have been particularly extensively used in the context of SARS-CoV-2 [45]. Our analysis, employing discrete phylogeographic methods, identified multiple introduction events of RSV into Russia. The majority of these were singletons, a pattern observed for both RSV-A and RSV-B. This suggests that individual introduction events frequently did not give rise to detectable transmission chains. A comparable result was reported for Kenya, where RSV-B dissemination was similarly attributable in large part to repeated importation events [46]. Studies conducted on SARS-CoV-2 also indicate that a predominance of singletons over cluster introductions is an expected outcome [47].

The introduction estimates obtained in the present study should be regarded as conservative, i.e., as lower bounds. Several factors account for this. First, the representation of complete RSV genomes from Russia in international databases remains limited. Although the number of whole genomes sequenced in the present study is comparable to the number already available in these databases, the overall count remains relatively small. In principle, a larger number of genomic sequences would enable the detection of a greater number of introduction events. Second, the counting methodology employed here records only the introduction event closest to the root along any given root-to-tip path in cases where multiple introductions are present, which further contributes to underestimation.

Nevertheless, the estimates obtained are robust. Varying the proportion of background (international) sequences had little effect on the results, indicating that the dataset included a sufficient number of such sequences. The estimates were likewise unaffected by the choice of phylogenetic reconstruction method: the use of the more computationally intensive maximum likelihood approach implemented in IQ-TREE 3 did not alter the results, nor did variation in the probability threshold applied for assigning internal nodes to different states [33].

Information on the immune status of the patients or their vaccination history was not available. Therefore, it was not possible to distinguish between two scenarios: virus spread in an immunologically naive population or the spread of newly emerging viral variants. We additionally tested whether introduction-associated branches carrying at least one reconstructed amino acid substitution in the F or G protein were more likely to give rise to cluster introductions than to singletons. No significant association was found for either subtype; however, the small number of introduction events limits the statistical power of this comparison, and the analysis addressed only the presence of any substitution rather than the effect of individual substitutions, which was beyond the scope of this study.

A limitation of this study is the restricted geographic coverage of the analyzed samples. Most sequences generated in this study originated from Moscow and the Moscow Region, while publicly available Russian sequences retrieved from GenBank or GISAID were either from Novosibirsk or were annotated only at the country level, without information on the specific region of origin. Such limited geographic sampling may bias the estimated number of introductions into Russia.

In the present dataset, substitutions associated with escape from nirsevimab or palivizumab were essentially absent. Only one RSV-B genome (retrieved from GISAID) carried F:N201S. These data can serve as a baseline for monitoring such variants after nirsevimab-based immunoprophylaxis is introduced in Russia.

## 5. Conclusions

In the present study, we performed whole-genome sequencing of RSV and conducted a phylogeographic analysis of virus introductions into Russia. Introduction events occurred repeatedly over time, and mostly were represented by single Russian sequences. This suggests that RSV introductions into Russia occur recurrently, but only a limited proportion of newly introduced viruses give rise to detectable transmission clusters.

## Figures and Tables

**Table 1:** Number of RSV-A viral introduction events estimated under different probability thresholds for assigning internal tree nodes to the “Russia” state and using different phylogenetic reconstruction methods. Values in bold are calculated via the primary method; adjacent columns represent validation data.

	FastTree, Threshold 0.6	FastTree, Threshold 0.7	FastTree, Threshold 0.8	IQTREE3, Threshold 0.7
Total introductions	46	**45**	44	45
Singletons	28	**28**	28	28
Cluster introductions	18	**17**	16	17

**Table 2:** Number of RSV-B viral introduction events estimated under different probability thresholds for assigning internal tree nodes to the “Russia” state and using different phylogenetic reconstruction methods. Values in bold are calculated via the primary method; adjacent columns represent validation data.

	FastTree, Threshold 0.6	FastTree, Threshold 0.7	FastTree, Threshold 0.8	IQTREE3, Threshold 0.7
Total introductions	39	**39**	37	39
Singletons	28	**28**	28	28
Cluster introductions	11	**11**	9	11

## Data Availability

Accession numbers for genome sequences generated in this study and used for phylogenetic analyses are provided in Supplementary Table S2. The sequences deposited in GISAID are available through the following EPI_SET: https://doi.org/10.55876/gis8.260804tw. All publicly available sequences retrieved from GenBank and GISAID and used in this study are listed in Supplementary Table S3. Additionally, we created EPI SET ID: EPI_SET_260709mh, https://doi.org/10.55876/gis8.260709mh.

## Supplementary Materials

The following supporting information can be downloaded at: https://www.mdpi.com/article/10.3390/v18080901/s1, Supplementary Table S1: Primers sequences; Supplementary Table S2: accessions for sequences generated in this study and used for phylogenetic analyses; Supplementary Table S3: GenBank and GISAID accessions for sequences used in this study; Supplementary File S1: GISAID Supplementary Appendix; Supplementary Table S4a: Comparison of the proportions of cluster and singleton RSV introduction events carrying at least one reconstructed amino acid substitution in the F or G protein; Supplementary Table S4b: Reconstructed amino acid substitutions in the F and G proteins on RSV-A introduction-associated branches; Supplementary Table S4c: Reconstructed amino acid substitutions in the F and G proteins on RSV-B introduction-associated branches; Supplementary Table S5. Nirsevimab and Palivizumab resistance-associated amino acid substitutions in the RSV F protein used for sequence screening.
