# Supplementary material for: Whole-Genome Sequencing of RSV and Phylogeographic Assessment of Viral Importations into Russia
Source: Viruses. 2026 Aug 15;18(8):901. doi: 10.3390/v18080901 (PMC13517720; doi:10.3390/v18080901)
Supplement: Supplementary file 1 [file viruses-18-00901-s001.zip › File S1.pdf]

Supplementary Appendix

All genome sequences and associated metadata supporting the findings of this study can be accessed through the persistent digital object identifier https://doi.org/10.55876/gis8.260709mh

In addition to the minted DOI, GISAID also communicates the aggregation of GISAID accession numbers (EPI_ISL_IDs) through the corresponding EPI_SET_260709mh identifier to facilitate both, the acknowledgment of all data contributors and the direct retrieval of the underlying data from GISAID used in this study.

RSV Data Summary

GISAID Identifier	Digital Object Identifier	Number of individual viruses	Data Collection range	Number of countries/territories
EPI_SET_260709mh	https://doi.org/10.55876/gis8.260709mh	2,406	2011-08-22 to 2025-12-24	58
